# Mathematical and intelligent modeling of stevia (*Stevia Rebaudiana*) leaves drying in an infrared‐assisted continuous hybrid solar dryer

**DOI:** 10.1002/fsn3.2022

**Published:** 2020-11-12

**Authors:** Adel Bakhshipour, Hemad Zareiforoush, Iraj Bagheri

**Affiliations:** ^1^ Department of Agricultural Mechanization Engineering Faculty of Agricultural Sciences University of Guilan Rasht Iran

**Keywords:** drying kinetics, infrared radiation, intelligent modeling, medicinal plant, solar energy

## Abstract

Drying characteristics of stevia leaves were investigated in an infrared (IR)‐assisted continuous‐flow hybrid solar dryer. Drying experiments were conducted at the inlet air temperatures of 30, 40, and 50°C, air inlet velocities of 7, 8, and 9 m/s, and IR lamp input powers of 0, 150, and 300 W. The results indicated that inlet air temperature and IR lamp input power had significant effect on drying time (*p* < .05). A comparative study was performed among mathematical, Artificial Neural Networks (ANNs), and Adaptive Neuro‐Fuzzy System (ANFIS) models for predicting the experimental moisture ratio (MR) of stevia leaves during the drying process. The ANN model was the most accurate MR predictor with coefficient of determination (R^2^), root mean squared error (RMSE), and chi‐squared error (χ^2^) values of 0.9995, 0.0005, and 0.0056, respectively, on test dataset. These values of the ANFIS model on test dataset were 0.9936, 0.0243, and 0.0202, respectively. Among the mathematical models, the Midilli model was the best‐fitted model to experimental MR values in most of the drying conditions. It was concluded that artificial intelligence modeling is an effective approach for accurate prediction of the drying kinetics of stevia leaves in the continuous‐flow IR‐assisted hybrid solar dryer.

## Graphical abstract

A comparative study was performed among mathematical, artificial neural networks (ANNs), and Adaptive Neuro‐Fuzzy System (ANFIS) models for predicting the experimental moisture ratio (MR) of stevia leaves during the drying in an infrared (IR)‐assisted continuous‐flow hybrid solar dryer. The drying time was decreased significantly by increasing the inlet air temperature and increasing IR lamp input power (*p* < .05). It was concluded that artificial intelligence modeling is an effective approach for accurate prediction of the drying kinetics of stevia leaves.

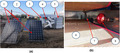

## 1 INTRODUCTION

Medicinal plants are regarded as rich resources of traditional medicines, and many of the modern medicines are produced from these plants (Dar et al., 2017). Stevia (*Stevia rebaudiana*), which is also known as candyleaf, sweetleaf, or sugarleaf, is a perennial plant of the family Asteraceae, originating from Paraguay. Stevia is a new emerging source of calorie‐free sweetener having no carbohydrate and fat (Ahmad et al., 2019; Marchyshyn et al., 2018; Singh & Rao, 2005). Stevia is a medicinal plant with a considerable demand in pharmaceutical, food and beverage industries (Gupta et al., 2011; Hajihashemi & Geuns, 2016) with increasing interest due to its health benefits, especially in diabetes and metabolic‐related disorders (López et al., 2016).

Drying is one of the most determining postharvest processes due to its advantages of inhibiting enzymatic degradation, microbial growth, and prolonging the shelf life (Oliveira et al., 2016; Wang et al., 2018). Drying is also the most popular method for preservation of medicinal plants. Appropriate drying of medicinal plants can increase the shelf life of medicinal plants by preserving their useful properties. Although low temperatures of between 30°C and 50°C are recommended by researchers for drying of medicinal plants, however, drying at such temperatures significantly prolongs the drying time and consequently decreases the capacity of drying instruments (Aboltins & Kic, 2016; Müller & Heindl, 2006; Sava Sand, 2015; Şekeroğlu et al., 2007). Several studies on the drying of plants were conducted by researchers (Aboltins & Kic, 2016; Arslan & Özcan, 2012; Bahammou et al., 2019; Bhardwaj et al., 2019; Moussaoui et al., 2020; Nadi & Abdanan, 2017; Venkatachalam et al., 2020). Téllez et al. (2018) conducted a research on the direct and indirect solar drying of stevia leaves. Their experimental results demonstrated the technical feasibility for the solar drying of stevia leaves. Experimental investigations on drying of stevia leaves have been carried out under mixed mode forced convection type (MFSCD) drying and open sun drying (OSD) methods by Lakshmi et al. (2019), and their results indicated that the solar‐dried stevia leaves provided better quality score compared to OSD samples. Lemus‐Mondaca et al. (2016) reported that drying temperatures of up to 50°C were appropriate for drying of stevia leaves and higher temperatures had negative effects on bioactive components, antioxidant capacity, and natural sweeteners of the stevia leaves.

Infrared (IR) drying is a combination of radioactive (at the surface of drying object) and conductive (inside of the object) heating technique in which the energy of IR waves is absorbed by the drying product, causing molecular vibration and in turn, simultaneously warming the surface and inner layers of the product (Doymaz, 2012; Nozad et al., 2016). IR spectra in the wavelength range of 2.5 to 200 μm are usually applied for drying purposes (Zare et al., 2015).

IR treatment has been reported to have many potential advantages over conventional drying methods such as higher drying rate, more energy efficiency, uniform or even product temperature, minimized losses and better quality of dried product, and less dust generation because of less airflow across the product (Lee, 2020; Sakare et al., 2020; Yadav et al., 2020). Furthermore, IR resource is less expensive in comparison with other new methods such as dielectric or microwave (Brandão et al., 2016).

Mathematical modeling of the drying kinetics of agricultural and food materials is a very important cognitive aspect that helps to have a better understanding and description about the drying behavior of the drying matter. Formulating the drying characteristics of foodstuffs is a very useful tool in the improvement of the design and the control of the drying process in the food industry (Tzempelikos et al., 2015). Mathematical modeling of thin layer drying of mint leaves in hot water recirculating solar dryer was carried out by Moradi et al. (2020) who reported that an approximation of diffusion model has the highest correlation with the experimental moisture ratio (MR) with coefficient of determination (*R* ^2^) of 0.98, root mean squared error (RMSE) of 0.041, and chi‐squared error (χ^2^) of = 0.0017. Mathematical modeling has been performed on the MR behavior of stevia leaves under a convective tray dryer. Experimental drying curves were modeled using eleven mathematical models, and the Midilli–Kucuk model was found to give the best fit quality with a *R* ^2^ of more than 0.99 (Lemus‐Mondaca et al., 2015).

Although mathematical modeling is a useful method to study the drying process of materials, but finding the best comprehensive mathematical model is difficult and time consuming, especially when there are several parameters affecting the drying process.

Intelligent modeling approaches such as Artificial Neural Networks (ANN) and Adaptive Network‐based Fuzzy Inference System (ANFIS) are being used increasingly in the agricultural and food studies. ANN is a well‐known technique that has been widely applied for simulation of drying kinetics of agro‐food products in several studies (Bai et al., 2018; Chasiotis et al., 2020; Tiwari, 2020). ANFIS is newer intelligent soft‐computing approach that employs the capabilities of neural networks and fuzzy inference systems. Several applications of ANFIS in food processing and technology were reported in a reviewed study conducted by Al‐Mahasneh et al. (2016). ANFIS is also successfully used to model the drying of foodstuffs (Ojediran et al., 2020; Prakash & Kumar, 2014).

A comparison between ANFIS, ANN, and mathematical modeling was applied by Kaveh et al. (2018) for predicting the drying characteristics of almond kernels in a convective dryer. It was reported that ANFIS had better prediction ability. It was also reported by Abbaspour‐Gilandeh et al. (2020) that the ability of ANFIS model was higher than ANN to predict the drying kinetics of quince slices in a hot‐air dryer.

A review of the literature showed that a comparative study has not been done to analyze the drying process of medicinal plants. Also, there are limited reports on the evaluation of medicinal plants drying in IR‐assisted continuous solar dryers. Therefore, the aim of this study was to investigate the drying behavior of stevia plant in a continuous dryer using the combination of direct and indirect solar systems along with solar‐powered IR radiation as auxiliary heating source.

## 2 MATERIAL AND METHODS

### 2.1 Drying apparatus

**Figure 1:**
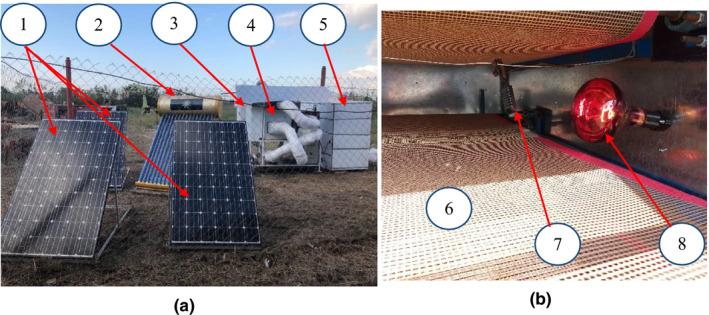
(a) Experimental setup of the drying system: (1) photovolaic solar panels; (2) solar water heater; (3) gas water heater; (4) gas–liquid heat exchanger; (5) drying chamber, (b) Interior view of drying chamber: (6) belt conveyor; (7) temperature and humidity sensor; (8) infrared lamp

In order to dry the product, an IR‐assisted continuous belt conveyor dryer was developed as the drying system (Figure 1a). The four‐floor continuous‐flow dryer was equipped with a hybrid of solar and gas water heaters, as main heating source of the system. A water pump was used to deliver water from water heater tank to a gas–liquid heat exchanger. The heat exchanger was of tube‐fin type with the liquid on the tube side. The ambient air from a centrifugal blower was passed through the heat exchanger and got the heat from the hot water current inside the pipes of the heat exchanger. The velocity of the inlet air to the drying chamber was adjusted using an inverter (LS, SV040iG5‐4, Korea) before the centrifugal blower. The temperature and relative humidity of the inlet air, and the air on the top of the samples, were monitored during the drying experiments using a temperature/relative humidity sensors (AM2301/DHT21, Model China). The inlet air velocity into the dryer was measured using a digital anemometer (Lutron Model YK, 80 Am, Taiwan). The comprehensive descriptions about the used heating system are presented in (Mehran et al., 2019). In this study, four solar‐powered infrared lamps with nominal power of 250 W (Noor lamp Co., Iran) were placed at the sides of the conveyor belts of the drying chamber (one lamp above each conveyor belt) for heating assistance (Figure 1b). The input power of IR lamps was adjusted using a rotational potentiometer and a digital multimeter (TES Model 232, Taiwan).

### 2.2 Sample preparation

During the experiments, the stevia leaf samples were collected daily from cultivated stevia plants in the research fields of the University of Guilan, Rasht, Iran. The Fresh leaves were carefully separated from the stems and poured into polyethylene bags until the drying process. The initial mass of the samples was 30 g which was measured using a digital balance with an accuracy of 0.001 g (A&D Model GX‐1000, Japan).

### 2.3 Drying conditions

Experiments were conducted between August and September of 2019, in the renewable energy site of the University of Guilan, Rasht, Iran. The IR‐convective drying experiments of stevia leaf samples were carried out in the continuous‐flow dryer at three levels of inlet air temperatures (30, 40, and 50°C), three levels of inlet air velocities (7, 8, and 9 m/s), and three levels of IR lamp input powers (0, 150, and 300 W). The experiments were carried out in three replications. In order to prevent leaf samples from physical damages and avoid losses during drying, the samples were carefully placed inside mesh bags and then the bags were positioned on the belt conveyor beds to be dried.

### 2.4 MR measurement and calculations

In order to determine the moisture content (MC) variations, the samples were weighed at the beginning of the experiments and at 15‐min intervals during the drying process. The samples were then kept at 103°C in an electric hot‐air oven for 24 hr (Cuervo‐Andrade & Hensel, 2016), to measure their dry matter content. The values of dry basis (d.b.) MC and MR of samples were calculated using the following equations (Ceylan & Gürel, 2016; Omolola et al., 2019):

(1) $${\text{MC}}_{d.b.}=\frac{m‐m_{\text{oven}}}{m_{\text{oven}}}$$

(2) $$\text{MR}=\frac{\text{MC}}{{\text{MC}}_{\text{in}}}$$

where ${\text{MC}}_{d.b.}$ is the dry basis moisture content, *m* is the mass of drying sample, and $m_{\text{oven}}$ is the mass of dry matter. Also, MC and ${\text{MC}}_{\text{in}}$ are the moisture content at any time and the initial moisture content, respectively.

### 2.5 Mathematical models

In order to find the most appropriate models for predicting the kinetics of drying stevia leaves, the experimental data of MR versus drying time were fitted with ten of the most common mathematical models (Babu et al., 2018; Ertekin & Heybeli, 2014; Kaveh et al., 2018; Onwude et al., 2016), (Table 1). The constants of the tested models were determined using the curve fitting toolbox of MATLAB programming software (MATLAB R2018b; The Mathworks Inc., Natick, MA, USA).

**Table 1:** Mathematical models used to describe the MR curves of stevia leaves, a, b, c, k, k1, and k2 are constants

Model name	Model Equation	Equation No.
Wang and Singh	$ax^{2}+bx+c$	(3)
Henderson and Pabis	$ae^{‐kx}$	(4)
Logarithmic	$ae^{‐kx}+c$	(5)
Approximation of diffusion	$ae^{‐kx}+\left(1‐a\right)e^{‐kbx}$	(6)
Page	$e^{‐kx^{n}}$	(7)
Two‐term	$ae^{‐k_{1}x}+be^{‐k_{2}x}$	(8)
Newton	$e^{‐kx}$	(9)
Simplified Fick's diffusion	$ae^{‐c(x/l^{2})}$	(10)
Modified Page equation‐II	$ae^{‐c{\left(x/l^{2}\right)}^{n}}$	(11)
Midilli and Kucuk	$ae^{‐kx^{n}}+bx$	(12)

### 2.6 ANN

**Figure 2:**
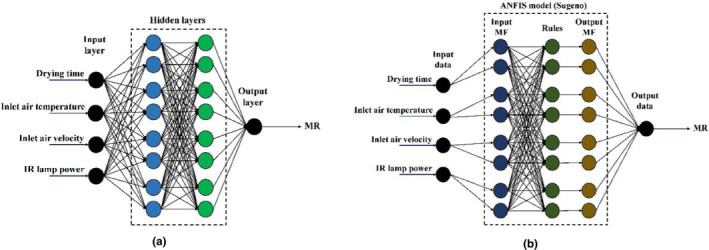
Schematic images of ANN structure with two hidden layers and eight neurons in each hidden layer (a) and ANFIS structure with two input MFs (b)

Manual development of a comprehensive model in the form of one equation for MR prediction so that it comprises all input variables of the drying conditions is complex and almost impossible. In order to include all of the input parameters in one predictive model, a feed‐forward multilayer perceptron ANN was developed in this study. Different structures of ANN were constructed and used for prediction of MR values during the drying process of stevia leaves. ANNs with one or two hidden layers, tangent sigmoid (tansig) or logarithm sigmoid (logsig) transfer functions, Levenberg–Marquardt (LM) or Scaled Conjugate Gradient (SCG) training techniques, and different neurons in the hidden layers (one to 20 neurons in each layer) were evaluated in this study. The pure‐line transfer function was applied to the output layer. The input data of ANNs were drying time, inlet air temperature, inlet air velocity, and IR power, while the MR values were the target data (Figure 2a). The dataset was randomly divided into three subsets including training (60%), cross‐validation (20%), and testing (20%) data. The most successful ANN was selected based on the highest value of *R* ^2^, and the lowest values of RMSE and χ^2^. Descriptions about these criteria are given in the following.

### 2.7 ANFIS

Different architectures of ANFIS were developed and employed for MR prediction. The input and target data were the same as those of ANN. There were four input variables, namely drying time, inlet air temperature, inlet air velocity, and IR lamp power. MR values were the target data. The initial datasets were also randomly splitted into 60% for training, 20% for validation, and 20% for testing.

The ANFIS toolbox of MATLAB software was used to build the ANFIS models. Totally, 36 different ANFIS structures were generated implementing grid partitioning technique by changing the parameters such as the number (two or three) of membership functions (MFs) in each input, the type of input MFs (Gaussian, Sigmoid, or Triangular), the optimization method (Backpropagation or Hybrid), and the type of output MF (Constant and Linear). A schematic image of ANFIS structure is shown in Figure 2b.

### 2.8 Statistical criteria

The predicted MR values of the developed models (mathematical, ANN and ANFIS) were compared to the experimental MR data based on three statistical criteria, namely *R* ^2^, RMSE and χ^2^ which were calculated using the Equations ((13), (14), (15)) (Qadri et al., 2020):

(13) $$R^{2}=1‐\left[\frac{\sum_{i=1}^{N}{\left({\text{MR}}_{exp,i}‐{\text{MR}}_{pred,i}\right)}^{2}}{\sum_{i=1}^{N}{\left({\text{MR}}_{exp,i}‐\bar{{\text{MR}}_{\exp}}\right)}^{2}}\right]$$

(14) $$\text{RMSE}={\left[\frac{1}{N}\sum_{i=1}^{N}{\left({\text{MR}}_{exp,i}‐{\text{MR}}_{pred,i}\right)}^{2}\right]}^{0.5}$$

(15) $$\chi^{2}=\frac{\sum_{i=1}^{N}{\left({\text{MR}}_{exp,i}‐{\text{MR}}_{pred,i}\right)}^{2}}{{\text{MR}}_{exp,i}}$$

where ${\text{MR}}_{exp,i}$ and ${\text{MR}}_{pred,i}$ are, respectively, the *i*th experimental and predicted MR data from N total MR values, and $\bar{{\text{MR}}_{\exp}}$ is the average of experimental MR values. The models with the highest R^2^ and the least RMSE and χ^2^ were selected as the most precise MR predictors.

## 3 RESULTS AND DISCUSSIONS

### 3.1 Drying kinetics and overall drying time

**Figure 3:**
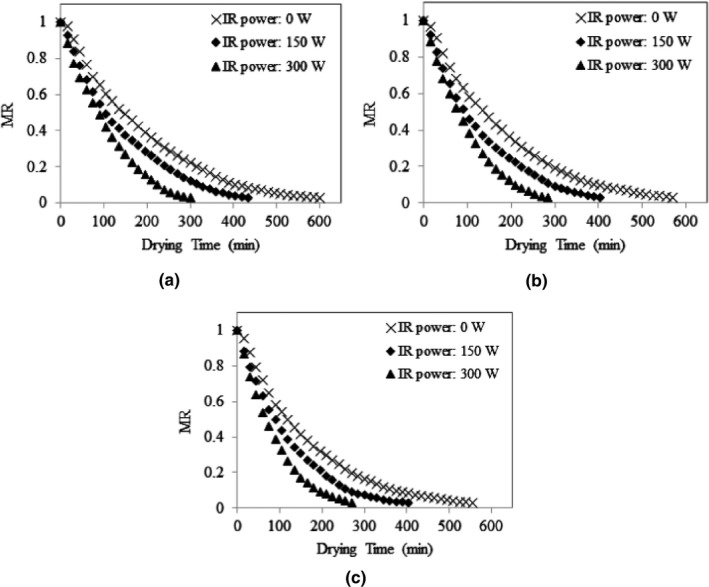
MR variations of stevia leaves under input air temperature of 30°C and different IR power values, at input air velocity of 7 m/s (a), 8 m/s (b), and 9 m/s (c)

**Figure 4:**
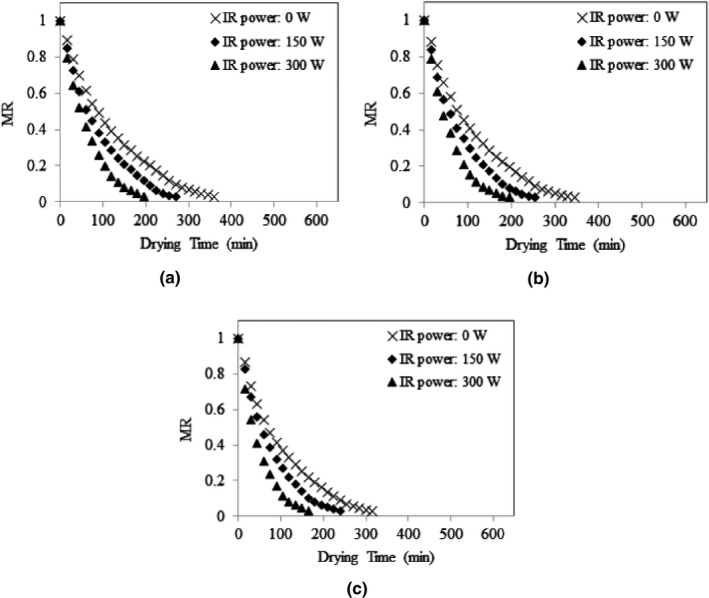
MR variations of stevia leaves under input air temperature of 40°C and different IR power values, at input air velocity of 7 m/s (a), 8 m/s (b), and 9 m/s (c)

**Figure 5:**
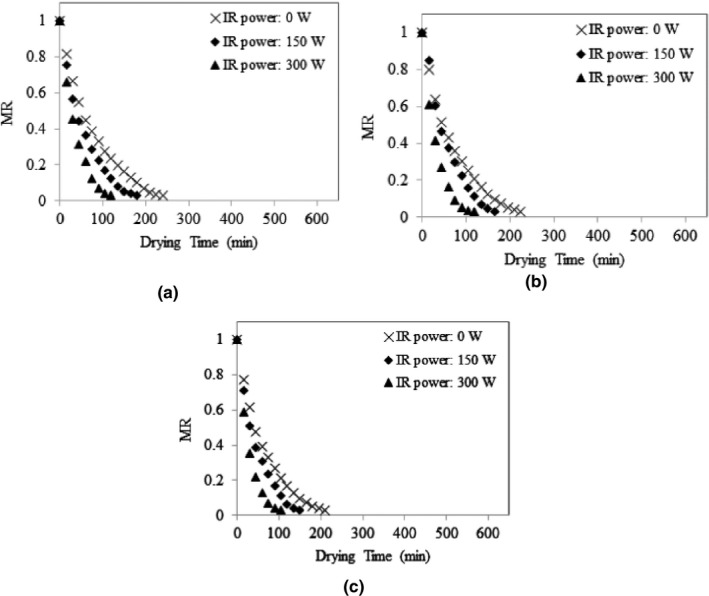
MR variations of stevia leaves under input air temperature of 50°C and different IR power values, at input air velocity of 7 m/s (a), 8 m/s (b), and 9 m/s (c)

The average initial moisture content of stevia leaves was 77.63 ± 0.1% w.b. (3.47 ± 0.02 d.b.) and the average final moisture content of the samples was 9.46 ± 0.44% w.b. (0.10 ± 0.01 d.b.), while the final MR was achieved around 0.03. Variations of MR values of stevia leaves under different drying conditions are presented in Figures 3, 4, 5. As shown, the MR values continually decreased by passing the drying time. The figures show that the drying rate was higher at early stages of drying process when the leaves had high moisture contents. The dehydration rate was reduced with decrease in the moisture content as the drying time was extended. Moreover, Figure 3(a) shows that, at the same inlet air temperature of 30°C, and the input air velocity of 7 m/s, the drying rate of stevia leaves increased by increasing the IR lamps power. Similar trends can also be observed in Figure 3(b,c) for inlet air temperature of 30°C, and the input air velocities of 8 m/s an 9 m/s; and in Figures 4 and 5 for inlet air temperatures of 40°C and 50°C. Increasing the drying rate of product by increasing the IR lamp power can improve the working capacity of the dryer. Although the IR lamps convert the electric power to infrared radiation which is an intensive energy‐demanding process, but in this study, this issue was resolved by employing photovoltaic panels to supply the power for IR lamps. Therefore, application of solar‐powered IR lamps can enhance the drying capacity of the system without any additional consumption of conventional energy sources.

It was observed that the air temperature and IR lamp power had a significant effect (*p* < .05) on the overall drying time. The overall drying time decreased by increasing inlet air temperature and IR lamp power. These results conform to those reported by Nozad et al. (2016) about the effect of drying temperature and IR radiation on the overall drying time of spearmint leaves and those revealed for thin‐layer infrared drying of mint leaves (Ertekin & Heybeli, 2014). The same results were obtained for some other products (Selvi, 2020; Younis et al., 2018; Zare et al., 2015).

**Figure 6:**
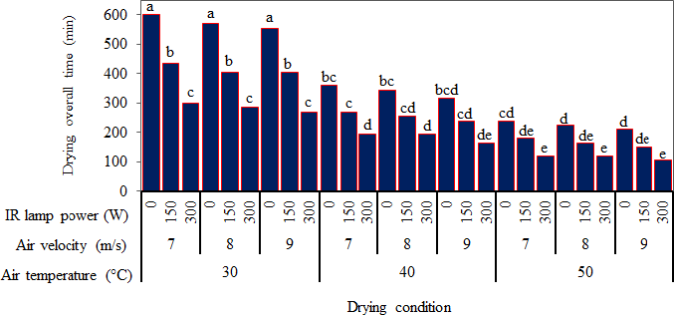
Averages of overall drying time in different drying condition. Different small letters indicate significant differences (*p* < .05) based on Duncan test

Figure 6 shows a graphical comparison of the overall drying time at different drying conditions, where the small letters above the bars indicate Duncan's multiple range test (*p* < .05). It can be seen in Figure 6 that for a given inlet air temperature of 30°C, and the input air velocity of 7 m/s, the overall drying time reduced significantly from 600 min to about half when the IR lamp power increased from zero to 300 W. Such a significant effect of IR lamp application was also observed for inlet air temperatures of 40 and 50°C. The increase of inlet air temperature also significantly decreased the overall drying time samples. In this study, it was observed that although the overall drying time was decreased by increasing the inlet air velocity, but its effect was not significant on the drying time. The overall drying time decreased from 600 min to 105 min when the drying condition was changed from inlet air temperature of 30°C, inlet air velocity of 7 m/s, and no IR radiation to an inlet air temperature of 50°C, inlet air velocity of 9 m/s, and IR power of 300 W.

### 3.2 Mathematical modeling

Different mathematical models according to Table 1 were evaluated for predicting MR variations of stevia leaf samples during the drying process. The statistical criteria and the constants of the best‐fitted models in each drying condition are presented in Table 2. It can be seen from this table that among all of the 27 drying conditions evaluated, the Midilli and Kucuk model gave the best results in 24 conditions. The Logarithmic model was the most effective model for MR prediction in two drying conditions of inlet air temperature of 30°C, inlet air velocity of 7 m/s, and IR power of 150 and 300 W. In the case of inlet air temperature of 30°C, inlet air velocity of 9 m/s and IR power of 300 W, the two‐term model was the best‐fitted one. It should be noted that the Midilli and Kucuk was the second effective prediction model in these three cases with very close statistical values. It was observed that in all cases, the Midilli and Kucuk model resulted in R^2^ of more than 0.998 which shows high competency of this model for describing the drying kinetics of stevia leaves. Midilli and Kucuk model was found to have the best fit for predicting of drying process of stevia leaves by Lemus‐Mondaca et al. (2015). This model was also reported applicable to describe the drying process of some other plant leaves such as savory (Arslan & Özcan, 2012), *Vernonia amygdalina* (Alara et al., 2018), peppermint (Torki‐Harchegani et al., 2016), *Asparagus officinalis* (Okur & Baltacıoğlu, 2018), and *Plectranthus amboinicus* (Nurafifah et al., 2018).

**Table 2:** The model constants and performance criteria of the best‐fitted models for prediction of MR variations during drying of stevia leaves in different drying conditions

T (ͦ C)	Air v (m/s)	IR. (W)	Best Model	Model statistic criteria			Model parameters				
				R^2^	χ^2^	RMSE	a	b	c	k	*n*
30	7	0	Midilli and Kucuk	0.9991	0.0031	0.0091	1.0160	1 × 10^–5^	–	0.0036	1.054
		150	Logarithmic	0.9993	0.0021	0.0077	1.0370	–	−0.0098	0.0059	–
		300	Logarithmic	0.9988	0.0022	0.0108	1.1130	–	−0.1151	0.0071	–
	8	0	Midilli and Kucuk	0.9992	0.0026	0.0086	1.0160	2 × 10^–5^	–	0.0038	1.064
		150	Midilli and Kucuk	0.9992	0.0018	0.0085	1.0120	−0.0001	–	0.0070	1.004
		300	Midilli and Kucuk	0.9996	0.0007	0.0065	0.9905	−0.0001	–	0.0047	1.138
	9	0	Midilli and Kucuk	0.9994	0.0019	0.0074	1.0190	1 × 10^–5^	–	0.0050	1.033
		150	Midilli and Kucuk	0.9996	0.0008	0.0057	0.9943	3 × 10^–5^	–	0.0062	1.046
		300	Two‐term	0.9997	0.0005	0.0057	−4.288	5.288	–	k1= 0.01737 k2= 0.01579	–
40	7	0	Midilli and Kucuk	0.9993	0.0014	0.0081	1.0060	−0.0002	–	0.0099	0.9408
		150	Midilli and Kucuk	0.9994	0.0010	0.0080	1.0040	−0.0002	–	0.0138	0.9329
		300	Midilli and Kucuk	0.9993	0.0010	0.0089	0.9932	−0.0001	–	0.0123	1.0420
	8	0	Midilli and Kucuk	0.9992	0.0016	0.0088	1.0050	−0.0002	–	0.0124	0.9128
		150	Midilli and Kucuk	0.9994	0.0009	0.0079	1.0020	−0.0002	–	0.0148	0.9410
		300	Midilli and Kucuk	0.9996	0.0005	0.0068	0.9973	0.0000	–	0.0131	1.0580
	9	0	Midilli and Kucuk	0.9991	0.0016	0.0092	1.0060	−0.0002	–	0.0148	0.8964
		150	Midilli and Kucuk	0.9995	0.0008	0.0075	1.0010	−0.0001	–	0.0137	0.9797
		300	Midilli and Kucuk	0.9994	0.0007	0.0086	0.9967	−0.0001	–	0.0236	0.9545
50	7	0	Midilli and Kucuk	0.9992	0.0011	0.0088	1.0040	−0.0002	–	0.0196	0.8889
		150	Midilli and Kucuk	0.9990	0.0012	0.0110	1.0010	−0.0003	–	0.0254	0.8962
		300	Midilli and Kucuk	0.9991	0.0008	0.0124	0.9987	−0.0003	–	0.0301	0.9519
	8	0	Midilli and Kucuk	0.9994	0.0009	0.0082	1.0020	−0.0002	–	0.0202	0.9009
		150	Midilli and Kucuk	0.9986	0.0015	0.0130	1.0020	−0.0002	–	0.0283	0.8923
		300	Midilli and Kucuk	0.9989	0.0010	0.0139	0.9979	−0.0002	–	0.0368	0.9403
	9	0	Midilli and Kucuk	0.9995	0.0007	0.0075	1.0010	−0.0002	–	0.0222	0.9044
		150	Midilli and Kucuk	0.9986	0.0015	0.0137	1.0010	−0.0003	–	0.0329	0.8687
		300	Midilli and Kucuk	0.9998	0.0001	0.0058	0.9998	−0.0001	–	0.0381	0.9677

### 3.3 ANN modeling

**Figure 7:**
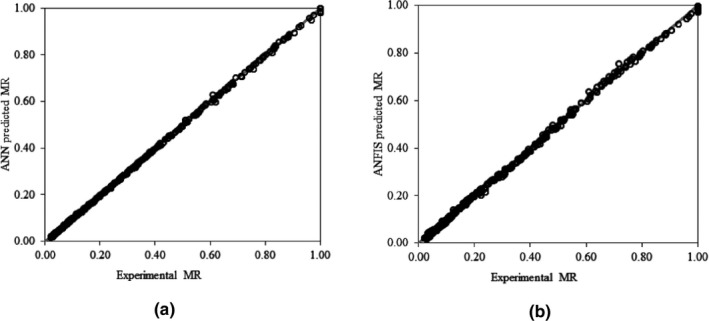
Experimental versus predicted MR values obtained from selected ANN (a) and ANFIS (b) models

Different architectures of ANN were constructed with one and two hidden layers, and different transfer functions and training algorithms were assessed for predicting the MR of stevia leaves. The optimum numbers of neurons in the hidden layers of these architectures were determined based on statistical criteria, and the most accurate topologies are listed in Table 3. In general, the ANNs with two hidden layers had better results than those with one hidden layer. Also, the ANNs with LM training algorithm were more accurate than those with SCG training algorithm. According to Table 3, among the evaluated ANN configurations, the two hidden layers neural network having 17 neurons in the first hidden layer and 19 neurons in the second hidden layer (4–17–19–1 topology), with logarithm sigmoid transfer function in the first hidden layer, tangent sigmoid transfer function in the second hidden layer, and LM error minimization algorithm was the most accurate ANN for monitoring the MR of stevia leaves during drying. This ANN model resulted in *R* ^2^ of 0.9999, RMSE of 0.0014, and χ^2^ of 0.0001 on the training dataset. The *R* ^2^, RMSE, and χ^2^ values of this ANN model on the test dataset were, respectively, 0.9995, 0.0005, and 0.0056, representing the high ability of the ANNs to reliably estimate the drying kinetics of stevia leaves in the IR‐assisted continuous hybrid solar dryer. Figure 7(a) shows the ANN predicted results versus experimental values of MR. The closeness of the data to the straight line with the slope equal to 1 shows the high prediction accuracy of the developed ANN technique. High capability of the ANN methodology has been also reported by Sarimeseli et al. (2014) for predicting the infrared drying behavior of thyme leaves, and other researchers who have used the ANN technique for modeling the drying kinetics of different crops (Bai et al., 2018; Khaled et al., 2020). The performance criteria of the selected ANN model were also better than those achieved by mathematical models. Higher MR prediction accuracy of ANNs compared to mathematical models was also reported by Karakaplan et al. (2019) for estimating the MR of spearmint during drying process, and by Omid et al. (2009) for modeling the drying kinetics of pistachio nuts. It should also be noted that the ANN enables us to easily provide a predictive model of the drying process for medicinal plants. Such ANN model can include all designated drying condition in a single structure.

**Table 3:** Topology and performance criteria of the most accurate ANNs for prediction of MR variations during drying of stevia leaves in different drying conditions

Training function	Transfer function of hidden layer(s)	Topology	Train			Test		
			*R* ^2^	χ^2^	RMSE	*R* ^2^	χ^2^	RMSE
**LM**	logsig	4‐17‐1	0.9995	0.0008	0.0063	0.9987	0.0020	0.0103
	tansig	4‐15‐1	0.9995	0.0007	0.0062	0.9984	0.0024	0.0115
	logsig ‐ logsig	4‐11‐17‐1	0.9999	0.0002	0.0015	0.9996	0.0006	0.0059
	**logsig ‐ tansig**	**4**‐**17**‐**19**‐**1**	**0.9999**	**0.0001**	**0.0014**	**0.9995**	**0.0005**	**0.0056**
	tansig ‐ logsig	4‐18‐20‐1	0.9999	0.0002	0.0017	0.9972	0.0052	0.0135
	tansig ‐ tansig sigmoid	4‐19‐14‐1	0.9999	0.0003	0.0030	0.9914	0.0286	0.0275
SCG	logsig	4‐14‐1	0.9907	0.0186	0.0268	0.9863	0.0215	0.0329
	tansig	4‐10‐1	0.9938	0.0063	0.0227	0.9928	0.0102	0.0236
	logsig ‐ logsig	4‐20‐11‐1	0.9979	0.0018	0.0135	0.9969	0.0063	0.0157
	logsig ‐ tansig	4‐16‐11‐1	0.9966	0.0029	0.0164	0.9941	0.0190	0.0228
	tansig ‐ logsig	4‐20‐19‐1	0.9991	0.0008	0.0083	0.9984	0.0032	0.0122
	tansig ‐ tansig **sigmoid**	4‐13‐13‐1	0.9989	0.0010	0.0099	0.9975	0.0058	0.0157

Bold highlight: The most accurate model.

### 3.4 ANFIS modeling

**Figure 8:**
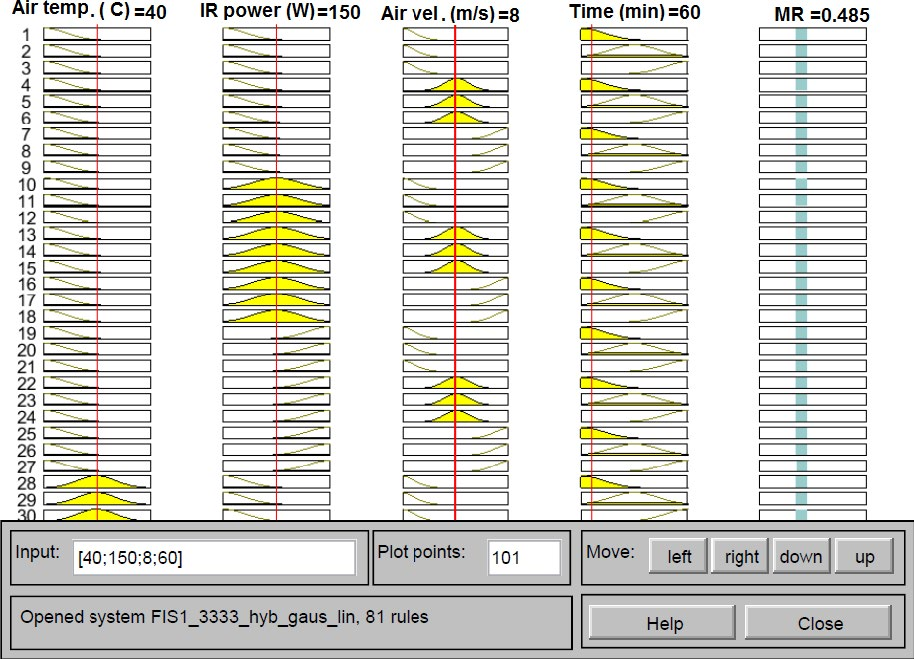
Rule viewer of the ANFIS model for the effect of the drying conditions on the MR of stevia leaves

The top six most efficient ANFIS models for MR prediction are presented in Table 4. According to the performance parameters, the ANFIS structure with Gaussian input MF, linear output MF, hybrid optimization method, and 3‐3‐3‐3 number of MFs resulted the highest prediction accuracy among all of the developed ANFIS models. The values of *R* ^2^, RMSE, and χ^2^ for this model on the training dataset were equal to 0.9994, 0.0068, and 0.0011, respectively. This model was evaluated on separated test dataset and yielded the *R* ^2^ of 0.9936, RMSE of 0.0243, and χ^2^ of 0.0202. These values showed that the ANFIS is an appropriate approach for monitoring the drying process of stevia leaves in IR‐assisted continuous hybrid solar dryer. Some of the ANFIS formulated rules are presented in Figure 8, the first 4 columns in this figure are ANFIS inputs and the fifth column shows the output of the structured ANFIS. It can be seen that for input values of 40°C inlet air temperature, 150 W IR power, 8 m/s air velocity and after 60 min drying time, the ANFIS predicted MR obtained was 0.485 which was equal to the average experimental MR of the latter drying condition.

**Table 4:** Structure and performance criteria of the most accurate ANFIS models for prediction of MR variations during drying of stevia leaves in different drying conditions

Number of MFs	Opt. Method	Input MF	Output MF	train			test		
				*R* ^2^	χ^2^	RMSE	*R* ^2^	χ^2^	RMSE
2‐2‐2‐2	Hybrid	Gaussian	Linear	0.9933	0.0122	0.0235	0.9910	0.0209	0.0288
2‐2‐2‐2	Hybrid	Sigmoid	Linear	0.9839	0.0195	0.0365	0.9828	0.0228	0.0396
2‐2‐2‐2	Hybrid	Triangular	Linear	0.9921	0.0163	0.0255	0.9911	0.0168	0.0285
**3**‐**3**‐**3**‐**3**	**Hybrid**	**Gaussian**	**Linear**	**0.9994**	**0.0011**	**0.0068**	**0.9936**	**0.0202**	**0.0243**
3‐3‐3‐3	Hybrid	Sigmoid	Linear	0.9991	0.0016	0.0087	0.9881	0.0357	0.0331
3‐3‐3‐3	Hybrid	Triangular	Linear	0.9975	0.0033	0.0145	0.9898	0.0192	0.0347

Bold highlight: The most accurate model.

ANFIS model was successfully used to predict the drying kinetics of quince slices during IR drying (Ziaforoughi et al., 2016). ANFIS was also reported to have the reliable ability to be applied in modeling and controlling of drying systems (Al‐Mahasneh et al., 2016).

As can be observed from Table 4, all of the most accurate ANFIS structures had a hybrid optimization algorithm and linear output MF, showing higher MR prediction capability of this arrangement over the other investigated combinations.

Comparison of the most accurate mathematical, ANN, and ANFIS models indicated that although all of these modeling methods are useful for predicting the drying kinetics of stevia leaves with high accuracy, but the ANN model was the most desirable model among the developed models, as it resulted the best performance criteria on training and test datasets. Figure 7 shows that the experimental versus ANN predicted data (Figure 7a) are less scattered around the diagonal line than the experimental versus ANFIS predicted data (Figure 7b). This proves that the neural network‐based model is a more accurate MR predictor than ANFIS model. The *R* ^2^, χ^2^, and RMSE values of the ANN model on the test dataset were 0.9995, 0.0005, 0.0056, respectively, which were superior than those obtained by ANFIS model (*R* ^2^ = 0.9936, χ^2^ = 0.020, and RMSE = 0.0243) on test dataset. Similarly, Lertworasirikul (2008) reported that the multilayer feed‐forward neural networks were slightly better than ANFIS and mathematical models for the prediction of MR of semi‐finished cassava crackers. Rad et al. (2018) reported that the ANN model was more accurate than fuzzy logic and mathematical model for prediction of white mulberry in infrared‐fluidized bed dryer. However, there are studies in which the ANFIS is reported as the most suitable model for MR prediction of drying matters, when compared to ANNs and mathematical models (Abbaspour‐Gilandeh et al., 2020; Kaveh et al., 2018). It should be noted that the accuracy of the ANFIS model could be improved by increasing the number of MFs; however, it drastically increases the number of rules. For example, when the number of input MFs is set to 2‐2‐2‐2 and 3‐3‐3‐3, the number of rules would be 16 and 81, respectively. Meanwhile the 4‐4‐4‐4 number of input MFs results in 256 rules. High number of rules would make the model too complex, and increases the convergence time of the model to the optimal solution (Alambeigi et al., 2016; Razin & Voosoghi, 2020).

## 4 CONCLUSION

In this study, drying behavior of stevia leaves in an IR‐assisted continuous‐flow hybrid solar dryer was investigated. Investigation of drying kinetics of stevia leaves showed that the falling rate of MR was increased by increasing input power of IR lamps and increasing the inlet air temperature. The overall drying time was significantly decreased by increasing the drying parameters of inlet air temperature and IR lamp power. Employing solar‐powered IR lamps in the continuous‐flow hybrid solar dryer significantly reduced the overall drying time which leads to improved dryer capacity with no excessive consumption of fossil energy. It can be concluded that the developed dryer system configuration in this study for stevia leaves drying has the advantages of high drying capacity and low usage of fossil energy.

Variations of MR values of stevia leaves were modeled using mathematical, ANN, and ANFIS models. It was observed that although all of these methods can effectively predict the drying kinetics of stevia leaves, but the ANN model was the most accurate with the best performance statistics. Besides their excellent predictive capabilities, ANN and ANFIS models, can be easily and simply trained to cover all drying conditions in one comprehensive model. These artificial neural learning techniques can be used for developing intelligent drying control systems with high reliability. The results of this study showed that the ANN model is the most suitable method for monitoring the IR‐assisted hot‐air drying process of stevia plant.

The applied dryer system and the proposed predictive model in this study, provide useful information toward the development of a clean, sustainable and intelligent technology for drying the medicinal plants.
